# A Community-Based, Mobile Electronic Medical Record System App for High-Quality, Integrated Antiretroviral Therapy in Lilongwe, Malawi: Design Process and Pilot Implementation

**DOI:** 10.2196/48671

**Published:** 2023-11-10

**Authors:** Caryl Feldacker, Raymond Mugwanya, Daniel Irongo, Daneck Kathumba, Jane Chiwoko, Emmanuel Kitsao, Kenn Sippell, Beatrice Wasunna, Kingsley Jonas, Bernadette Samala, Daniel Mwakanema, Femi Oni, Krishna Jafa, Hannock Tweya

**Affiliations:** 1 Department of Global Health, University of Washington Seattle, WA United States; 2 International Training and Education Center for Health Seattle, WA United States; 3 Medic Kampala Uganda; 4 LifeNet International Kampala Uganda; 5 Lighthouse Trust Lilongwe Malawi; 6 Medic Nairobi Kenya; 7 Medic San Francisco, CA United States; 8 International Training and Education Center for Health Lilongwe Malawi; 9 Medic Abuja Nigeria

**Keywords:** antiretroviral therapy, differentiated service delivery, digital innovations, Malawi, mobile electronic medical record systems, monitoring and evaluation

## Abstract

**Background:**

Differentiated service delivery (DSD) increases antiretroviral therapy (ART) access in sub-Saharan Africa by moving clients out of congested ART clinics to communities for care. However, DSD settings challenge provider adherence to complex, chronic care treatment guidelines and have burdensome systems for client monitoring and evaluation (M&E), reducing data for decision-making. Electronic medical record systems (EMRS) improve client outcomes and reduce M&E workload. Traditional EMRS cannot operate in most DSD settings with unreliable power and poor connectivity.

**Objective:**

This study aims to detail the human-centered design (HCD) process of developing a mobile EMRS for community-based DSD services in Lilongwe, Malawi.

**Methods:**

Lighthouse Trust (LT) operates 2 Ministry of Health (MoH) clinics in Lilongwe, Malawi, with a combined total of >35,000 ART clients. LT’s real-time, point-of-care EMRS collects complex client M&E data and provides decision-making support, ensuring adherence to integrated HIV and tuberculosis guidelines that optimize client and program outcomes. LT’s EMRS scaled to all large MoH ART clinics. LT also implements a nurse-led community-based ART program (NCAP), a DSD model to provide ART and rapid assessment for 2400 stable LT clients in the community. LT, alongside collaborators, from the University of Washington’s International Training and Education Center for Health and technology partner, Medic, used the open-source Community Health Toolkit (CHT) and HCD to develop an open-source, offline-first, mobile EMRS-like app, “community-based ART retention and suppression” (CARES). CARES aims to bring EMRS-like provider benefits to NCAP’s DSD clients.

**Results:**

CARES design took approximately 12 months and used an iterative process of highly participatory feedback sessions with provider, data manager, and M&E team inputs to ensure CARES optimization for the NCAP and LT settings. The CARES mobile EMRS prototype supports NCAP providers with embedded prompts and alerts to ensure adherence to integrated MoH ART guidelines, aiming to improve the quality of client care. CARES facilitates improved data quality and flow for NCAP M&E, aiming to reduce data gaps between community and clinic settings. The CARES pilot demonstrates the potential of a mobile, point-of-care EMRS-like app that could benefit NCAP clients, providers, and program teams with integrated client care and complete M&E data for decision-making. CARES challenges include app speed, search features to align longitudinal records, and CARES to EMRS integration that supports timely care alerts.

**Conclusions:**

Leveraging the CHT and HCD processes facilitated the design of a locally specified and optimized mobile app with the promise to bring EMRS-like benefits to DSD settings. Moving from the CARES prototype to routine NCAP implementation should result in improved client care and strengthened M&E while reducing workload. Our transparent and descriptive process shares the progress and pitfalls of the CARES design and development, helping others in this digital innovation area to learn from our experiences at this stage.

## Introduction

Antiretroviral therapy (ART) increases survival and quality of life for people living with HIV. As of 2022, a total of 20 million people were on ART, and several countries in sub-Saharan Africa attained the Joint United Nations Programme on HIV/AIDS (UNAIDS) 95-95-95 targets [1]. Despite these gains, ever-increasing numbers of clients with ART translate into higher health care costs, crowded facilities, and prolonged client wait times, ultimately reducing care quality. To address some of these challenges, the World Health Organization (WHO) recommends differentiated service delivery (DSD)—a person-centered approach that simplifies and adapts HIV services across the care cascade in ways that serve the needs of clients with ART while optimizing the available resources in health systems [2]. DSD reduces client burdens by moving stable clients from crowded public clinics to community care for less intensive, lower-cost services [2,3]. DSD reduces congestion at the ART facility clinic and may improve access to ART services [3]. However, routine, comprehensive client monitoring and evaluation (M&E) needed to deliver high-quality HIV care is challenged by community settings [4,5]. In community DSD, health care workers (HCWs) typically rely on burdensome, paper-based reporting for client management and program M&E [6]. With poor M&E, DSD may increase the risk of loss to follow-up, delay viral load (VL) monitoring, or miss the timely identification of low-level viremia [7-9]. Innovations that provide high-quality M&E to optimize ART care in DSD settings without adding HCW workloads are a worldwide priority [10].

Electronic medical record systems (EMRS) improve ART client care by helping providers act in accordance with guidelines, improving client outcomes [11]. EMRS also manages high data volumes and streamlines reporting, reducing workload [12]. However, after 20 years of EMRS investment, EMRS is implemented in only 15% of low-income countries [13]. Typical EMRS operate in hospitals and require consistent electricity, digital literacy, and connectivity. Even with expanding cell service and internet, fewer than 30% of sub-Saharan African health care settings have reliable power [14,15]. EMRS are rare in public sub-Saharan African clinics, especially low-resource DSD settings where the majority of ART clients seek care [16]. Without EMRS, most DSD providers cannot adhere to complex, integrated M&E guidelines [17] that optimize client outcomes.

Mobile health (mHealth), the use of mobile phones or other wireless technology, offers a nimble bridge to bring EMRS benefits to DSD. mHealth innovations are recommended by WHO [18] and used widely in low-resource settings to strengthen ART adherence, retention, and VLs [19-22]. mHealth can interoperate with and expand the reach of EMRS to streamline data capture, cleaning, management, and routine analytics [23]; increase efficiency for reduced workload [24]; and lower costs [25,26]. Unlike conventional EMRS, mHealth is faster to design, simpler to deploy, cheaper to maintain, and can operate without constant connectivity or electricity [27]. Supporting community DSD with an mHealth EMRS innovation could improve clinical oversight and integrated care in communities, decreasing the burden on public and specialty clinics. Translating promising mHealth to public DSD settings in ways that synchronize with EMRS and reduce workload could realize intended DSD benefits for client-centered care and for clinic decongestion.

In Malawi, Lighthouse Trust (LT), a WHO-recognized center of excellence, implements a DSD approach, the nurse-led community ART program (NCAP), specifically designed for stable ART clients [28]. In NCAP, nurses provide ART services to clients through peer support meetings in the community. Recent NCAP assessment found high satisfaction and a desire to expand. The 2 primary weaknesses limit NCAP scale-up. First, LT’s EMRS works only in its clinics with a consistent network and power, not in community settings where NCAP operates. Without EMRS, NCAP clients receive less comprehensive ART care than their static-site peers. Second, without EMRS, NCAP M&E is burdensome; that is, NCAP nurses enter visit data into tablets and print it for manual EMRS entry by clerks. NCAP M&E is inefficient, incomplete, and error-prone, compromising effective NCAP and LT program management.

Therefore, in 2021, in direct response to NCAP nurse needs [28] and worldwide calls for evidence on DSD client outcomes [29,30], LT, the International Training and Education Center for Health (I-TECH), and Medic collaborated to combine mHealth advantages with EMRS benefits, co-designing a tablet-based, mobile, point-of-care (POC) EMRS app for NCAP—community-based ART retention and suppression (CARES). CARES leverages an open-source, worldwide-good Community Health Toolkit (CHT), building CARES on the existing CHT core framework. CARES aims to address several persistent weaknesses in mHealth [31-33]: (1) NCAP nurses are LT nurses who comfortably use EMRS in clinic settings and CARES in NCAP, showing high digital literacy; (2) clinical reviews last 7 minutes using EMRS, reducing workload concerns; (3) iterative, highly participatory, human-centered design (HCD) processes inform CARES local specification and optimization in line with best practices for HCW and Ministry of Health (MoH) buy-in; and (4) CARES extended EMRS-supported care to NCAP, meeting expressed provider and MoH needs.

For CARES, we combine HCD with robust implementation science methods to document the process from design through implementation in alignment with WHO best practices for digital health M&E [34]. In this first paper, we focus on CARES HCD processes co-led by LT’s NCAP and M&E teams in close partnership with Medic and I-TECH. We describe the CARES CHT framework, system development process, and CARES features as we move from prototype launch for NCAP clients from LT’s 2 urban flagship clinics (Lighthouse clinic [LH] at Kamuzu Central Hospital and Martin Preuss Centre [MPC] at Bwaila Hospital) and prepare to expand across all NCAP settings. Transparent sharing of the process, pitfalls, and progress of CARES HCD aims to inform other digital health implementers of our success and lessons learned, with the hope of enhancing collaboration to bring mobile EMRS to reality in routine DSD settings.

## Methods

### Setting

The CARES app was developed for the LT’s NCAP program at its 2 MoH flagship clinics in urban Lilongwe, Malawi, with a combined total of >35,000 ART clients: 24,000 at MPC and 11,000 at LH. As part of MoH services, LT also supports care at 7 rural and periurban satellite sites in Lilongwe district. LT service providers, including nurses and M&E teams, work across clinic settings, caring for clients across facilities and locations. Like other large Malawi ART clinics, all LT facilities, including both LH and MPC, use a real-time POC EMRS at its static sites to ensure adherence to MoH integrated ART guidelines that optimize client ART care and program outcomes [35] and ease routine M&E reporting [36]. The EMRS is scaled to all large- and medium-volume MoH ART clinics in Malawi [37]. The EMRS are stand-alone and do not synchronize client data between clinics. NCAP services are offered to clients from both LT flagship clinics as well as 7 satellite health facilities.

### Description of the NCAP

To access NCAP services, a client must meet the following criteria: (1) be an adult aged 18 years or older; (2) be on a first- or second-line ART regimen for a minimum of 6 months; (3) be deemed “stable” by an ART provider; (4) show no severe side effects or opportunistic infections; (5) demonstrate viral suppression or undetectability; (6) reside within the catchment areas of the Lighthouse Community Health Services (NCAP); and (7) be a member of an ART peer support group. NCAP services are offered where ART peer support groups meet for psychosocial support. NCAP services reflect client-centered care and have a unique hierarchical care model; clients are referred from 9 LT facilities (2 flagship and 7 satellite) and mixed within 120 peer community groups that are served by NCAP nurses from the flagship sites. Clients in the same support group meet monthly. Not all NCAP clients receive ART or a clinical review at each meeting but are scheduled for a 3-6–month ART supply depending on clinical considerations. NCAP clients who need referrals for specialty care (cervical cancer screening, tuberculosis treatment initiation, family planning initiation, or evaluation for potential ART switch) return to their static referral facility for care. NCAP clients typically do not transfer between static referral sites but maintain their clinic record at one facility while receiving most of their care in the NCAP setting.

### Description of Current NCAP Data Collection and Management

Before an NCAP visit, NCAP nurses prepare a list of clients due for appointments in a specific peer support group, as well as those eligible for VL sample collection and preparing a list of HIV commodities (eg, ART, antihypertensive drugs, VL sample tubes, and contraceptives). During the clinic review, the NCAP nurse verifies the client’s identification, conducts clinical assessment, including ART adherence calculations, and enters the data into a tablet using a data management form called the Open Data Kit (ODK). Clients with good ART adherence are given a new ART supply, while those with poor ART adherence are referred to their static ART clinic for intensive ART adherence counseling. The ODK data are printed for retrospective data entry in the respective client EMRS by data clerks.

The NCAP ODK-based app has several weaknesses that CARES aims to address. First, as the ODK-based app does not use national IDs for client identifiers, clients may be registered with different names at different health facilities, leading to duplication of client records. Also, the ODK-based app lacks decision-making support for providers, which is crucial for complex, integrated ART management. Nurses use a combination of paper and an ODK-based app for lab sample reminders and results management, potentially resulting in an increased workload for providers. The ODK-based app does not track drug movements across health facilities, leading to inconsistent stock management across multiple NCAP referral facilities. Printing NCAP ODK records for manual data entry into EMRS is work-intensive. Overall, while benefiting clients, NCAP’s model of client-centered care creates multiple provider and program challenges that grow increasingly complex as more clients and facilities want NCAP services.

### CHT Software Technology and Infrastructure

The CARES app was developed using the CHT, an open-source project stewarded by Medic [38]. The CHT is a robust platform designed to create digital health apps that are client-centered and suitable for low-resource settings. The CHT leverages a set of popular, tested, and proven software technologies, including CouchDB, XLSform(), and postgres. The core framework comes built-in with nontrivial modules that most public software solutions need to address, such as security, roles and permissions, reporting hierarchies, tasks, and scheduling. The CHT also includes configurable forms, data fields, and analytics across 5 main feature sets included in CARES: longitudinal client records, task management, decision support, direct provider-to-client messaging like 2-way text messaging, and analytics. By providing these out-of-the-box capabilities, the CHT saves significant time and resources, allowing app developers to focus on customizing the app to meet their project needs.

The CHT’s architecture is designed to achieve horizontal scalability, making it suitable for large-scale deployments. CHT has been designed to conform to a range of interoperability standards, including OpenHIE, OpenMRS, and HL7. In order to achieve contextual adaptability, digital health apps built using the core framework support multiple languages, including English, French, Hindi, Nepali, Spanish, Swahili, and Indonesian, with new languages added in the admin console. CHT apps run offline-first and work with basic feature phones (through SMS text messages), smartphones (Android app), tablets, and computers. CHT apps use a common codebase on all devices. By using the same source code across all platforms, digital health tool kits ensure a consistent user experience regardless of whether the app is accessed from a desktop or mobile device. This unified approach not only reduces barriers for users but also simplifies maintenance for developers by eliminating the need for separate codebases.

Medic supports CHT users with a mature code base, provides robust technical assistance, and fosters a large and fast-growing open-source community. Apps powered by the CHT now support more than 48,000 frontline health workers in 16 countries in Africa and Asia as they perform over 100 million caring activities to date. CHT is recognized as a digital worldwide public good [39].

### CARES App Development Process: HCD

**Figure 1:**
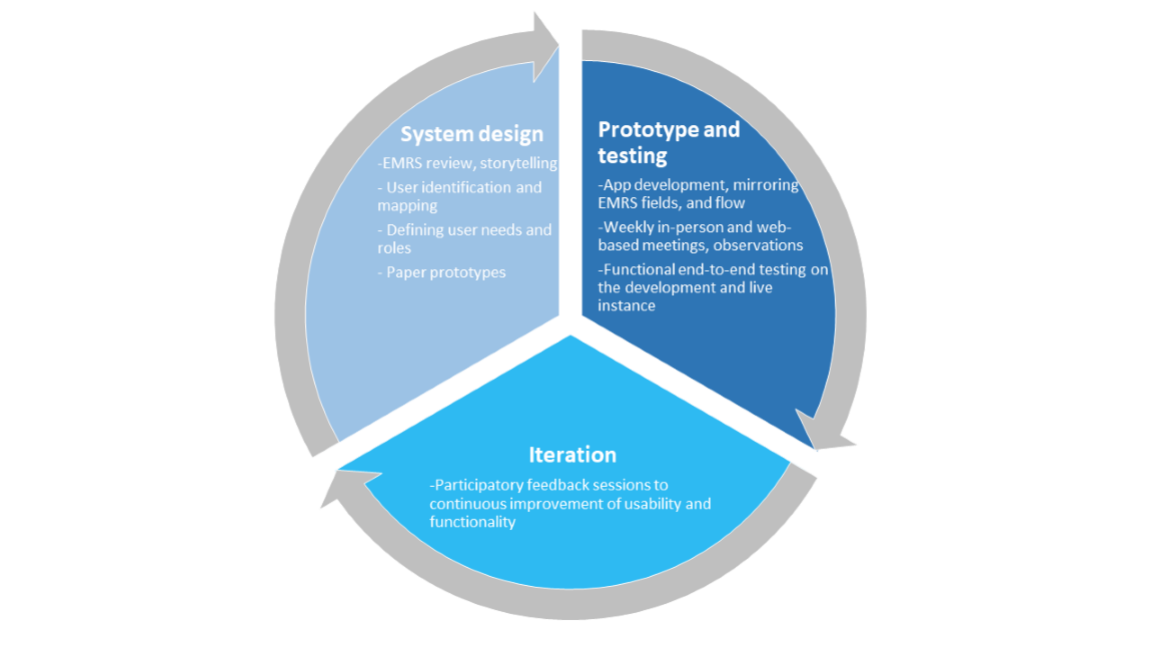
Key human-centered design components for community-based antiretroviral therapy retention and suppression (CARES) app iterations. EMRS: electronic medical record system.

Medic, I-TECH, and LT collaborated as a multidisciplinary team to cocreate the development of the CARES app. HCD was used to promote consistent engagement of HCWs, incorporate an iterative process of diverse feedback, and facilitate the creation of contextually relevant technology. Medic’s HCD process has been described in detail previously [40-45]. We describe 3 core HCD phases of the CARES app development: system design, prototype testing, and iteration (Figure 1).

### System Design (4 Months)

As the Malawi ART program uses a well-designed and functional EMRS, the CARES app acts as an extension of the EMRS in the community. Subsequently, the development team reviewed the existing EMRS flows, features, and data storage as well as observed ART nurses in static facilities to understand the users’ needs, behaviors, and challenges. The system development team also conducted informal interviews and used storytelling techniques with M&E staff, NCAP nurses, and ART nurses in LT flagship clinics. After understanding the NCAP process and functional and nonfunctional requirements for CARES, the team identified users and mapped the users to roles in NCAP services and program data management (Table S1 in Multimedia Appendix 1). The system design process identified areas for improvement (Table 1), which guided the overall goal of developing the CARES system.

**Table 1:** Description of current nurse-led community-based antiretroviral therapy program (NCAP) monitoring using Open Data Kit (ODK), community-based antiretroviral therapy retention and suppression (CARES) app features, and expected CARES advantages.

Service component	Current NCAP standard	NCAP with CARES	CARES advantages
Client treatment history	No previous treatment data available	Some clients’ visit records held locally on tablets	Previous information on the latest viral load, tuberculosis, side effects, allergies to sulfur, family planning methods, ART^a^ regiment, and appointment date
Complete client assessment	Abridged assessment adherence	Comprehensive, integrated EMRS^b^-like ART visit	Complete, high-quality M&E^c^ data for all NCAP clients. Provider decision-making supports ensure adherence to integrated care guidelines. CARES embedded alerts flag poor adherence, hypertension, viral load monitoring, side effects, and referrals
Management of NCAP ART drugs	Nurses pull paper files. Collect and manually reconcile ART drugs	Drug collection forms ease	Automated estimation of drugs needed for NCAP visit and drugs remaining after the NCAP visit
NCAP data to clinic EMRS	Data from ODK are extracted, printed, and manually entered in the EMRS	Offline CARES data synchronized to static EMRS	Daily CARES to EMRS synchronization reduces data errors and workload. Provides timely data for program monitoring

^a^ART: antiretroviral therapy.

^b^EMRS: electronic medical record system.

^c^M&E: monitoring and evaluation.

Although the CARES data flow and fields mirror the EMRS, the CARES system was designed in alignment with the appearance and interface of the CHT. During the iterative co-design process with HCWs, including NCAP nurses and M&E teams, LH teams learned the CHT system’s interface, its “alerts” and “tasks” features, and how to interact with it correctly. LT HCWs repeatedly engaged in cycles of observing, testing, interacting, and providing design feedback with the aim of making CARES app more acceptable, usable, and useful for the HCWs. At each cycle, user feedback was incorporated into CARES system layout, navigation, and client flow. The resulting CARES co-design process reflected LT user optimization within the structural confines of CHT-based CARES. At the culmination of a 6-month intensive process of weekly meetings and user sessions, LT’s NCAP nurse, data, and M&E users reported that CARES was intuitive, easy to use, and visually appealing.

### Prototype Testing (4 Months)

The first functional prototype of the CARES app took approximately 4 months to build. As intended, CARES largely mirrored the EMRS fields and flow, with a CHT user interface. Weekly web-based and in-person system review meetings were conducted with NCAP nurses who participated in the testing process, pushing mock clients through the CARES app to mimic integrated ART care in the NCAP setting. NCAP nurses repeated testing functionality and features, gaining confidence in managing client flows through the logical visit prompts, until they achieved competence. Users also tested, identified gaps, and provided inputs to improve decision support, stock management, and data flows, resulting in CARES prototype changes that delayed sharing but improved contextual matching. The CARES prototype was also presented to MoH staff for feedback, allowing time for subsequent improvements to security and flow in accordance with MoH’s evolving policy.

### Iterations and Piloting (4 Months)

HCD is an iterative, highly participatory feedback and optimization process. Biweekly prototype feedback sessions (1 for theoretical and brainstorming and 1 for hands-on practice) from October 2022 to January 2023 continued to inform CARES prototype evolution. The multidisciplinary team conducted weekly web-based and in-person meetings to review system development progress. The development team continuously refined, revised, and ultimately improved the prototype, gradually and repeatedly enhancing both the usability and functionality of the CARES app. The iterations and piloting aim to improve CARES usability, characterized by how users, in this HCD case, HCWs, consider the usefulness, simplicity, and benefit of digital innovation [46,47]. Usability is a core component of HCD.

From January to March 2023, the CARES team undertook a 3-month CARES pilot (with a concurrent ODK-based app as a backup) process over several distinct steps. First, the complete CARES prototype was hosted on the development instance, a secure space without client data that facilitated rigorous, complex testing from nurses and program leads. The local hosting of the prototype allowed for a safe environment to put the CARES system through a cyclical, prelaunch testing process to approximate pressure for a highly responsive app to create a seamless client experience. Once satisfied with the results of the iterative prototype review, a 1-day, hands-on orientation and training session was conducted for NCAP nurses and M&E officers to confirm CARES minimal viable product acceptability and usability before launch. Additional feedback from NCAP staff further informed software modification, stability enhancements, and EMRS fidelity in preparation for full implementation. The CARES app was launched in a beta phase in June 2023.

### Ethical Considerations

The CARES study protocol was approved by the Malawi National Health Sciences Research Committee (protocol 21/11/2830) and the University of Washington, Seattle, United States (STUDY00013936) ethics review board. No identifiable data were used in this design documentation. No consent was sought or obtained for this formative manuscript. Participants who provided information on app design for this manuscript were not compensated in any way.

## Results

### The CARES App

CARES is an offline-first, real-time, POC ART client management app that mimics the static ART EMRS while providing additional features to improve ART client care in the community. The app operates on Android tablets. When logged in, NCAP nurses access CARES components in the same order as the content of the EMRS, including NCAP registration, anthropometric measurements, VL monitoring, ART clinical review, drug dispensing, and automated appointment scheduling. The CARES app captures complete ART clinic review data, providing real-time benefits, including access to historical ART data and clinic decision support for integrated HIV care that helps ensure adherence to MoH ART guidelines. CARES forms, mandated fields, and verification checks ensure complete data, while external routine quality assurance confirms quality data before merging with EMRS data. The CARES app uses independent servers at LH and MPC with Representational State Transfer application programming interface services that integrate with EMRS and synchronize data at specific intervals, maintaining a record of all changes made over time.

### NCAP Services Using the CARES App

**Figure 2:**
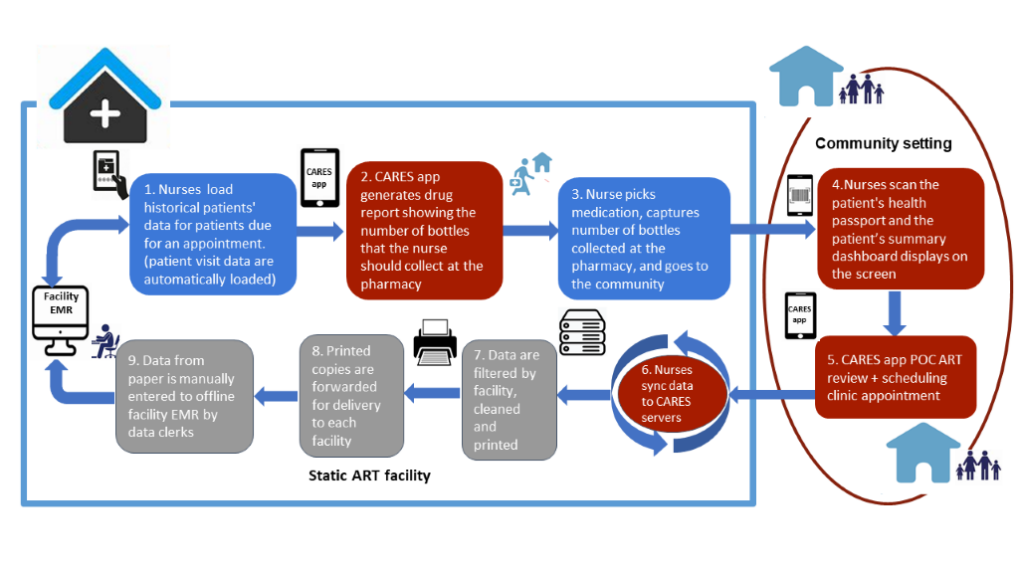
Step-by-step process of nurse-led community-based antiretroviral therapy program (NCAP) nurse activities guided by the community-based ART retention and suppression (CARES) app in both clinic and community settings. ART: antiretroviral therapy; EMRS: electronic medical record system; POC: point-of-care.

**Figure 3:**
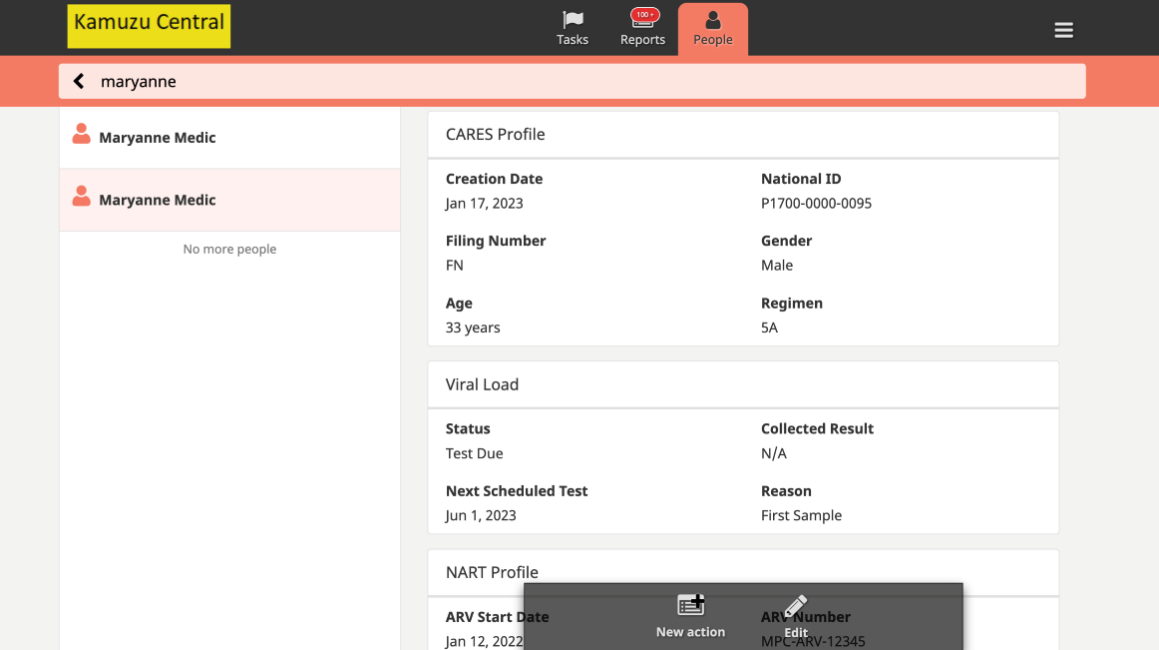
Community-based antiretroviral therapy retention and suppression (CARES) app’s individual-level client dashboard as viewed on each client’s People page.

CARES streamlines NCAP client flow for nurses (Figure 2). When a nurse logs into CARES at a static ART facility (LH or MPC), the latest ART visit data for all NCAP clients is automatically loaded, providing users with up-to-date information to aid in ART client care. The app generates a drug supply report, including the number of people due for appointments and those eligible for annual VL sample collection. In the community, nurses search for and confirm the client ID, pulling up the client summary dashboard with key clinical care data from the POC EMRS dashboard (Figure 3). The CARES app then guides nurses through a complete POC ART review, including reception, vitals, clinic consultations, adherence, prescription, drug dispensing, and scheduling an ART review appointment. CARES improves care and client M&E by mirroring EMRS modules, question flow, embedded prompts, and decision support. After providing NCAP services, the nurse visits the ART clinic to synchronize the data with the EMRS. The NCAP nurse connects the tablet to the Wi-Fi network and opens the CARES Android app, which automatically initiates the data synchronization process. However, in some cases, the nurse may need to manually synchronize the data by clicking on the “Sync Now” button.

### CARES Features and Functionality

#### Optimized for Offline-First (Low Connectivity) Settings

The CARES app is designed to be used in settings with limited internet connectivity by using the offline capabilities of the CHT through PouchDB and CouchDB. First, the CHT provides offline storage for data through PouchDB, enabling health care providers to access client records and other information even when an internet connection is not available. Second, CHT offline forms enable health care providers to collect client data even when there is no internet connection. Lastly, CARES app stores users’ data locally on their device so that workflows can be completed without synchronizing to the server. During offline periods, nurses can access or make changes to data locally on the device and synchronize the data with the central server when connected to the network at the ART clinic. CouchDB provides server-based storage and replication.

#### Improved Client Verification

During NCAP enrollment, health care providers scan the barcode sticker on the client’s health passport in lieu of manually typing in a client’s health ID, importing the client ID number directly into the CARES ID form field and removing the potential to incorrectly enter ID numbers or letters. Health passports are rarely lost or unreadable. The barcode scan feature is incorporated into the CARES app through the tablet’s camera, creating a more accurate and efficient entry of client ID.

#### Adherence to Integrated HIV Guidelines

**Figure 4:**
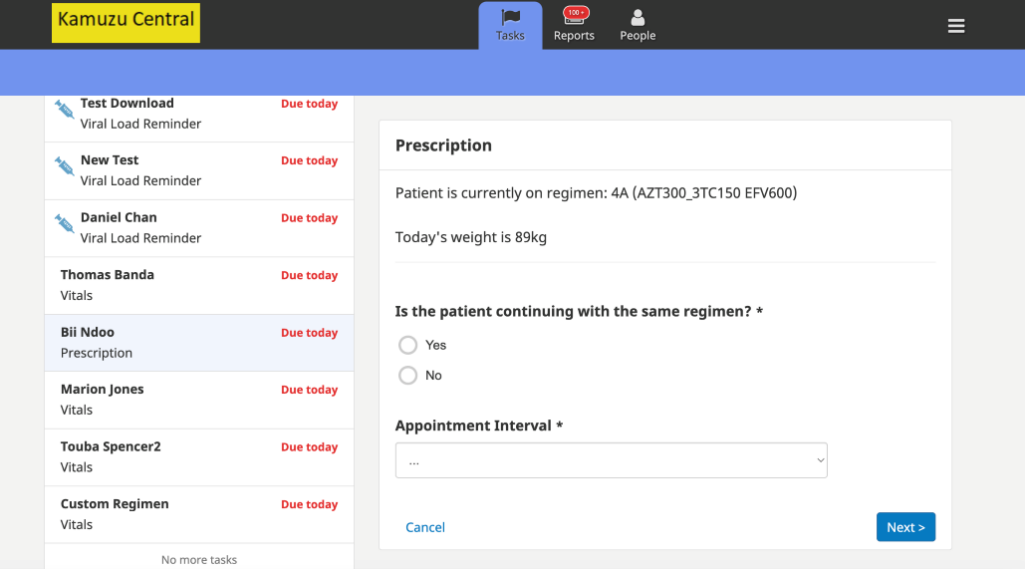
Community-based antiretroviral therapy retention and suppression (CARES) app’s individual-level prescription page.

In the absence of clinic-based data collection systems, such as EMRS, NCAP providers faced challenges in adhering to complex, integrated HIV guidelines or providing the standard minimal health care package to optimize client outcomes. The CARES app aimed to improve NCAP care quality and efficiency by incorporating EMRS-like clinical decision support. In addition to the system using historical data to guide visit forms and fields, providers can also access previous ART visit records on the client summary. The app alerts nurses to ART adherence, retention in care, and ART side effects. The app-based alert system also reminds NCAP nurses of the annual VL test, facilitating the provision of timely and appropriate care to clients. CARES calculates client ART adherence based on consideration of drugs received at the previous visit and the number of tablets brought to the current review, determining if the client requires enhanced adherence counseling. CARES confirms maintenance of the same ART regimen or allows for updating (Figure 4).

#### Improved On-Time Annual VL Monitoring

**Figure 5:**
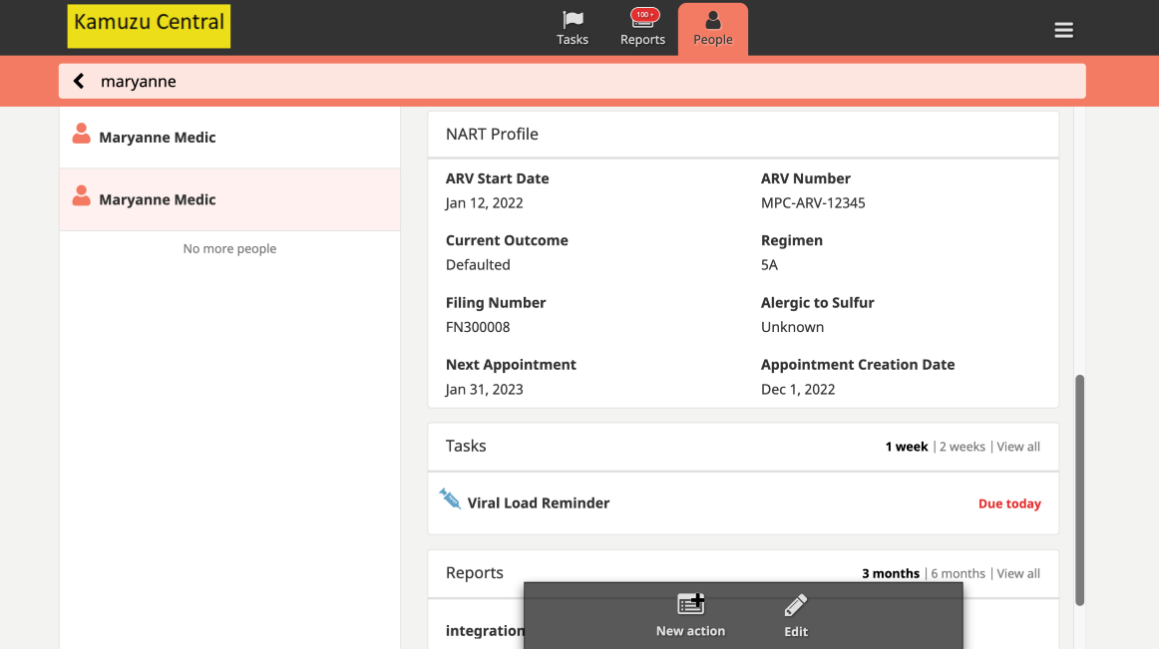
Client profile with an example of viral load reminder in the community-based antiretroviral therapy retention and suppression (CARES) app.

Timely monitoring of VL is important to detect treatment failure early and to help identify individuals who may need a change in their ART regimen. In addition, VL screening is used to monitor ART adherence and identify clients who need additional support. Determining the appropriate time for collecting annual VL in large ART cohorts can be a complex process; missing annual VL monitoring can lead to delayed detection of treatment failure and an increased risk of HIV transmission. As part of improving on-time VL monitoring, the CARES built-in algorithms aim to alert providers to take the annual VL sample when it is due. The app also displays the latest VL results and any new test ready to share with the client (Figure 5).

### CARES Security Features

The CARES app incorporates multiple robust security features to safeguard client data, ensuring the confidentiality and privacy of client information. First, on the tablets, CARES enforces the use of password-protected user accounts. CARES users are required to create strong passwords to access the system, reducing the possibility of unauthorized access. The CARES app has full disk encryption and an auto-lock screen for Android phones. Role-based access further restricts data to ensure that different users have different levels of access to client data based on their position and responsibilities. This allows only authorized users to view and modify client data, reducing the risk of data leaks. For data transmission and storage, the CARES app provides secure transfers through perfect forward secrecy and 4096-bit SHA-2 certificates by default. To further enhance security, the app uses a nonstandard port for Secure Shell access, which reduces the risk of automated brute-force attacks. Additionally, CARES accepts only public key authentication for Secure Shell connections, providing an extra layer of protection against unauthorized access. The system maintains an audit trail of all user activity, including data access, modification, and deletion, allowing for easy monitoring and detection of any suspicious activity. Lastly, the system complies with local data protection laws and regulations, ensuring that client data are handled responsibly and ethically.

### CARES Usability

Since the inception of the process, 11 HCWs, including nurses, have participated in HCD sessions. By August 2023, data from 2084 NCAP clients had been successfully imported into CARES, and 6 nurses had completed ART reviews for 361 clients registered at LH and 355 from MPC. Of all client records, 2.9% (21/716) were incomplete and required manual data management.

## Discussion

### Overview

The HCD approach was used to create an open-source, offline-first mobile EMRS app prototype, CARES, with expected benefits across 2 critical aspects of service delivery: improved DSD client care and reduced HCW workload. The app aims to provide mobile, POC, EMRS-like advantages to DSD settings, supporting integrated, high-quality client care and quality data to inform program M&E. CARES mimics the decision support of EMRS, reflects the usability priorities of nurse users, and focuses on short-term improvements in urban sites, both LH and MPC. The CARES system directly responds to expressed NCAP nurse needs and worldwide calls for evidence on DSD client outcomes. CARES leverages an open-source, worldwide-good CHT. Building CARES on the existing CHT core framework saved time and increased its potential for scale. CARES’ design process implemented core characteristics of mHealth excellence, including optimizing HCW accessibility and acceptance, appropriate technology, local adaptation, strong stakeholder collaboration, and government partnerships [48]. We discuss lessons learned and the next steps for the CARES system implementation and evaluation.

The use of HCD during the development of the CARES app provided several advantages. First, CARES gained practical implementation optimization evidence for future expansion within NCAP. By optimizing CARES+ in the routine NCAP context, CARES+ prioritizes worldwide emphasis on improving client ART care, strengthening data quality, timely collection, and use in routine program settings [49,50]. Second, CARES promoted stakeholder engagement from inception by involving diverse HCW groups (nurses, NCAP managers, M&E teams, and data managers) throughout the development process, which ensured that their perspectives and insights were incorporated into the CARES prototype. This collaborative approach heightened interest and excitement in the process and resulted in an app that reflected the needs of the nurses in these community-based DSD settings. HCD also promoted contextually relevant technology by considering the local factors and constraints, testing, and revising the app to meet the low connectivity settings rather than expecting that the connectivity would improve to meet mHealth hardware or software needs. In line with HCD processes, CARES considers local infrastructure, resources, and service delivery norms, increasing the likelihood of successful adoption and sustained use.

The CARES prototype encountered, documented, and mapped solutions for common mHealth challenges. Like other mHealth innovations, even with offline-first priority, connectivity is a constraint. NCAP nurses are still required to physically visit each satellite facility to connect to the clinic intranet to transfer the NCAP data to the static EMRS. CARES cannot yet bring M&E workload advantages to satellite sites. Second, the NCAP program’s distinct client-centered approach to service delivery is challenging for the traditional app hierarchy. The CHT core framework requires a hierarchical structure to organize client information in the app. Typical hierarchies reflect the health system, health program, or community service delivery structures and most commonly have clients nested under fixed facilities, providers, or location groupings. NCAP does not work this way. NCAP clients come from 9 referral facilities and mingle in mixed support group settings to receive community-based care from an LT nurse from a flagship site. Before and after visits, data must come from and return to referral clinic’s EMRS. The NCAP groups are associated with multiple referral ART clinics, creating a many-to-many association that is not supported by traditional health system hierarchies. In response, and due to CHT core framework constraints, the design team was faced with unique challenges to adjust the CARES app to conform with fixed CHT hierarchy structures. Ultimately, CARES configuration aligned clients with a facility first, and then with an NCAP group as an attribute of the client record. In this way, CARES had to adjust to the CHT constraints instead of redesigning the CHT, adding unexpected time and iteration to app design and development. Lastly, CARES+ design and monitoring align with the WHO digital health maturity life-cycle [34], moving from assessment of functionality, stability, and fidelity to consistency. This is not a quick or inexpensive process. Deliberate, iterative, and highly participatory HCD slowed the pace of CARES design while also increasing the resources needed for development. These time and additional resource requirements could make this type of HCW process less accessible in other low-resource settings. However, it is expected that these intensive investments will now pay long-term dividends, resulting in a CARES app that is optimized for the NCAP setting with the high levels of buy-in needed to make it a sustained success.

### Conclusions

CARES aims to bring EMRS benefits to DSD settings, meeting the expectations and needs of nurse-led HIV care in community settings. As CARES moves from prototype to practice, several critical improvements are underway to fulfill the promise of CARES for DSD clients, providers, and programs. First, CARES requires optimization of data storage. As with any app, there is a limit to how much data can be stored locally, particularly on a mobile device. For users needing access to large amounts of data without slowing down the speed of services, data purging rules can ensure that users can access the necessary data without taking up too much space on their devices. Second, CARES needs to incorporate noncommunicable disease management, like hypertension, into the NCAP setting. CARES’s next iterations will include the EMRS hypertension module to improve the quality of NCAP client care, again aiming to provide similar care quality in both clinic and community settings. Moreover, CARES features must facilitate improved client referrals between NCAP and static ART clinics. These strengthened referrals would better address the needs of NCAP clients with noncommunicable diseases, high VLs, or low-level viremia, streamlining the continuum of HIV care and follow-up while enhancing the accuracy of data to address care gaps along the cascade. Lastly, strengthened EMRS integration could further automate data extraction and reduce workload while improving data use for NCAP program evaluation. This improved data access would facilitate data use by program administrators, help identify client- and program-level gaps, and monitor improvements. Improved data access could also allow for more efficient data analysis and reporting, streamlining dissemination efforts. As CARES moves from prototype to implementation in NCAP routine practice, rigorous evaluation and frequent dissemination will bring important learnings for digital innovation practitioners and policy makers in the near future. Additional investments in CARES and similar digital health efforts are warranted.

## Appendix

Multimedia Appendix 1 NCAP users and their roles.

## Abbreviations

ART: antiretroviral therapy
CARES: community-based antiretroviral therapy retention and suppression
CHT: Community Health Toolkit
DSD: differentiated service delivery
EMRS: electronic medical record systems
HCD: human-centered design
HCW: health care worker
I-TECH: International Training and Education Center for Health
LH: Lighthouse clinic
LT: Lighthouse Trust
M&E: monitoring and evaluation
mHealth: mobile health
MoH: Ministry of Health
MPC: Martin Preuss Centre
NCAP: nurse-led community-based antiretroviral therapy program
ODK: Open Data Kit
POC: point-of-care
UNAIDS: Joint United Nations Programme on HIV/AIDS
VL: viral load
WHO: World Health Organization

## Data Availability

Data sharing is not applicable to this article as no data sets were generated or analyzed during this study on the CARES app design process and pilot test.
