# Supplementary material for: A Community-Based, Mobile Electronic Medical Record System App for High-Quality, Integrated Antiretroviral Therapy in Lilongwe, Malawi: Design Process and Pilot Implementation
Source: JMIR Form Res. 2023 Nov 10;7:e48671. doi: 10.2196/48671 (PMC10674144; doi:10.2196/48671)
Supplement: Multimedia Appendix 1 [file formative_v7i1e48671_app1.docx]

**Table S1.** NCAP users and their roles.

| Key Personas | Activities |
| --- | --- |
| Patients | - Visit NCAP meeting point every 3-6 months for scheduled appointment - Bring health passport booklet to NCAP - Bring ART and CPT remaining pills to NCAP for adherence check - Visit his/her clinic at any point necessary for clinical consultations |
| Community Health Services nurse (NCAP nurse) | At referral static clinics   - Collect ARVs, CPT, INH and Pyridoxine from referral clinics for expected NCAP patients, including buffer stock (quarterly) for transport and storage at Lighthouse central NCAP distribution point - Request and document other drugs and supplies from Lighthouse pharmacies (condoms, scales, and IEC materials, etc) - Reconcile all drugs and supplies at the end of each quarter   At Lighthouse before visiting NCAP settings   - With M&E support, nurse is provided with a prints a list of patients that are due for NCAP appointment in the EMR. - Prior to clinic visits, a NCAP nurse generates a list of patients due for appointments in specific distribution points as well as those eligible for VL sample collection to prepare. - Prepare documentation of Viral Load samples to be collected - Gather routine commodities required for the community ART services - Collect Viral Load results from facility data clerks and communicate to respective patients in the community   In the community NCAP settings   - See appointed patients at the outreach location one-on-one; - Complete an abridged ART visit using open data kit (ODK) entering patient-level data into the tablet   - Adherence monitoring (pill count)   - Refill of prescriptions   - Documentation of current status on patient passport and patient chart (that will later be entered into EMR)   - Provision of condoms   - Provision of additional family planning methods (pill, depot)   - Screening for comorbidities such as tuberculosis, hypertension, etc.   - Follow-up treatment of hypertensive cases that are already diagnosed and   - treatment prescribed by a clinician. - Referral to clinic for:   - Moderate or severe HTN for review and treatment initiation   - Diabetes   - Cervical cancer screening/FP implants   - High viral load for intensive adherence counselling   - Screening for comorbidities such as tuberculosis, hypertension, cervical cancer screening, etc.   - Imaging such as CXR, ultrasound etc - Dispense 3-6 months’ supply of prescription pills to patients seen - Draw plasma viral load samples from eligible patients - Issuing of next appointment date at the outreach location   At Lighthouse after completing community visits:   - Transport all VL samples to Lighthouse laboratory - Return tablet to Lighthouse static sites M&E team |
| Data Entry Clerk/M&E personnel | - Enter data retrospectively with vital signs, drug dispensed, next appointment date from Lighthouse NCAP database into the facility EMR. - Check and collect Viral Load results from blood draw area - Update Viral Load register at the facility - Link up with NCAP nurse on Viral Load results received - Print visit summary and stick them on patients charts in collaboration with receptionists |
